# Supplementary material for: The interactome of intact mitochondria by cross-linking mass spectrometry provides evidence for coexisting respiratory supercomplexes
Source: Mol Cell Proteomics. 2017 Dec 8;17(2):216–32. doi: 10.1074/mcp.RA117.000470 (PMC5795388; doi:10.1074/mcp.RA117.000470)
Supplement: Supplemental Data [file supp_RA117.000470_133922_0_supp_23978_fzffwf.zip › spectra_annotation/mito_DR_spectra_annotation/176-1-7-1-2-1.pdf]

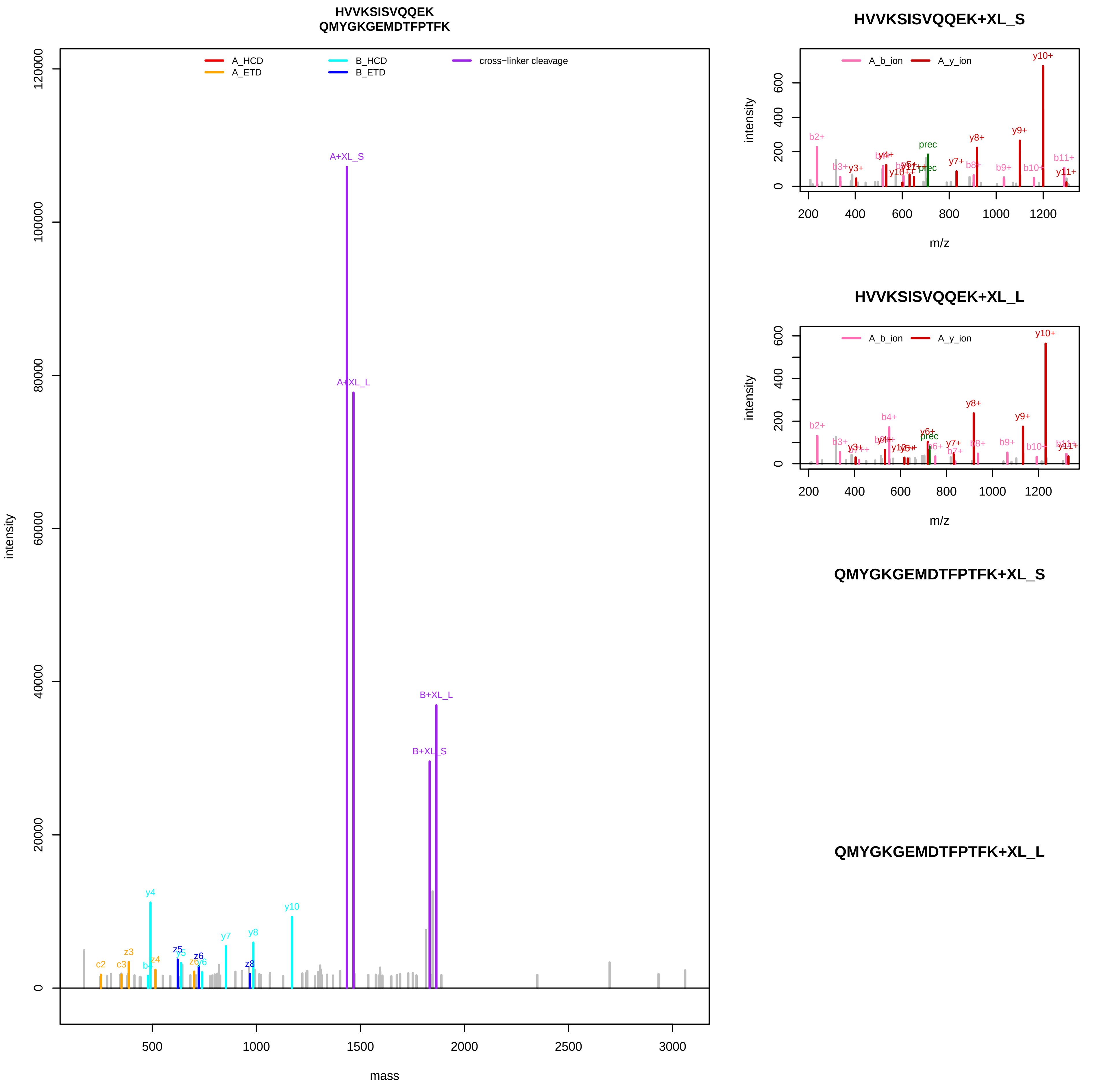
HVVKSISVQQEK
QMYGKGEMDTFPTFK
A_HCD
A_ETD
B_HCD
B_ETD
cross−linker cleavage
A+XL_S
A+XL_L
B+XL_L
B+XL_S
y4
y10
y7
y8
z5
z6
z8
c2
c3
z3
z4
intensity
mass
HVVKSISVQQEK+XL_S
A_b_ion
A_y_ion
y10+
y9+
y8+
y7+
b2+
b3+
y3+
prec
b9+
b10+
b11+
y11+
m/z
HVVKSISVQQEK+XL_L
b4+
y6+
QMYGKGEMDTFPTFK+XL_S
QMYGKGEMDTFPTFK+XL_L
